# To be or not to be a Queen: the dark side of the ant genome

**DOI:** 10.1186/1756-8935-6-S1-P55

**Published:** 2013-03-18

**Authors:** Claire Morandin, Yutaka Watanabe, Misato Okamoto, Man Ying Tin, Alexander Sasha Mikheyev

**Affiliations:** 1 Department of Biological and Environmental Science, Centre of Excellence in Biological Interactions, University of Helsinki, P.O. Box 65,00014, Helsinki, Finland; 2 Okinawa Institute of Science and Technology, Ecology and Evolution Unit, Okinawa 904-2234, Japan

## Background

Understanding how dissimilar phenotypes are produced from a similar set of genes is a crucial topic in evolutionary biology. In social insects, both workers and queens develop from diploid eggs, and the extent of phenotypic dimorphism between castes varies between species. Gene expression differences underlying the two female castes, queens and workers, are crucial for social insect evolution. However, it is unknown how much the caste biased proportion of the genome varies in quantity or quality and how this relates to the level of caste polymorphism. This project investigates the differences in gene expression between female castes in *Wasmannia auropunctata* using deep transcriptional sequencing to understand the role of coding, and potentially non-coding sequences on caste differentiation.

## Materials and methods

We used samples originating from an invasive population on the Big Island of Hawaii (USA) and native populations from Kourou, French Guiana (France). The reference genome was constructed using the Hawaiian population. Using the sum total of proteins predicted by the other ant genome projects, and 886,838,537 mapped reads, automatic annotation was then carried out using the MAKER2 pipeline. For the gene expression analysis, we used RSEM software to map transcripts originating from 14 different sets of libraries.

## Results

The genome assembly contained 103, 611 contigs (N50 37, 912 bp). The RNA seq data contained 73,798 unique transcripts from 25,594 expressed genomic loci. Of these loci 10,457 had homology to other known proteins and no protein predicted by the pipeline. Of the transcripts without protein predictions, 6,306 showed significant differential expression between the female castes. To identify IncRNA, we filtered these transcripts to remove any residuals protein coding genes, ribosomal RNA and other kind of coding RNAs, and evaluated the proteing coding potential of these transcripts using CPC.

## Conclusions

In ants, phenotypically distinct queens and workers develop from similar genomes. Therefore, the morphological, physiological and behavioural differences are likely to be based on differential gene expression. To date, the mechanisms behind caste differential expression in ants are largely unknown. In our study species we identified hundreds of possible IncRNA with differential expression between queens and workers. These new findings strongly suggest a correlation between differential gene expression from female caste and the prevalence of IncRNA. Their characterization and expression is a novel starting point for the study of reproductive division of labor in social insects. Further studies would need to investigate whether those differentially expressed IncRNA are conserved among insect species to establish stronger evidence that IncRNA play a role in caste differentiation in social insects.

